# Ambient Aqueous-Phase Synthesis of Copper Nanoparticles and Nanopastes with Low-Temperature Sintering and Ultra-High Bonding Abilities

**DOI:** 10.1038/s41598-018-38422-5

**Published:** 2019-01-29

**Authors:** Yoichi Kamikoriyama, Hiroshi Imamura, Atsushi Muramatsu, Kiyoshi Kanie

**Affiliations:** 1 Tohoku University, Institute of Multidisciplinary Research for Advanced Materials, Sendai, 980-8577 Japan; 2 Mitsui Mining & Smelting Co., Ltd., Corporate Engineered Materials Sector R&D Center, Ageo, 362-0021 Japan

## Abstract

Copper nanoparticles (NPs) with an average particle diameter of 50–60 nm were successfully obtained by reducing an aqueous solution of a copper(II)-nitrilotriacetic acid complex with an aqueous hydrazine solution at room temperature under an air atmosphere. Copper NP-based nanopastes were printed onto a glass substrate using a metal screen mask and pressureless sintered under a nitrogen atmosphere at 200 °C for 30 min. The electrical resistivity of the resulting copper electrode was 16 μΩ · cm. For a metal-to-metal bonding test, copper nanopaste was printed on an oxygen-free copper plate, another oxygen-free copper plate was placed on top, and the bonding strength between the copper plates when pressureless sintered under a nitrogen atmosphere at 200 °C for 30 min was 39 MPa. TEM observations confirmed that highly crystalline metal bonding occurred between the copper NPs and the copper plate to introduce the ultrahigh strength. The developed copper NPs could provide promising advances as nanopastes for sustainable fabrication of copper electrodes and die attachment materials for the production of next-generation power semiconductors.

## Introduction

Copper electric circuits on substrates in smart devices, such as tablet PCs and mobile phones, are widely manufactured by a photolithography process. This process is advantageous for fabricating large-scale, high-quality integrated circuits. However, the multistaged masking and etching process requires expensive production systems and the use of harmful and environmentally undesirable chemicals. On the other hand, printed electronics (PE) technology^1–6^ has attracted a great deal of attention as an alternative eco-friendly and low-cost process to write electric circuits on flexible substrates with metal inks. Recently, a combination of PE and integrated circuit (IC) production technologies, flexible hybrid electronics (FHE)^2–11^, is a promising technology for flexible and wearable smart devices because the FHE-based on-demand and eco-friendly process allows the production of a wide variety of devices based on market needs. For practical use of PE and FHE technologies to fabricate on-demand devices, bonding materials that are applicable to flexible substrates are needed. Currently, solder alloys and conductive epoxy polymers have been widely applied as materials. On the other hand, metal inks and pastes have considerable potential as bonding materials to fabricate next-generation power semiconductors (SiC and GaN) and as alternatives to solder alloys for electronic parts in automobiles^12–14^. Nano- and micron-sized silver particles have been widely utilized in inks and pastes due to the high electric and thermal conductivity and low-temperature sintering property of silver. For example, intermetallic bonding with silver paste used for sintering is carried out at a low temperature of 300 °C or less, and the obtained bond strength between metals reaches 30 MPa or more^15–19^. To ensure the operational stability of next-generation power semiconductors and automotive electronic components, which are expected to be used in high-temperature environments (200 °C), intermetallic bonding materials are required to have high heat dissipation, high thermal resistance, and low resistance^20–22^. The thermal conductivity of bulk silver is 420 W·m^−1^·K^−1^, and the advantage of silver has resulted in a focus on applications as nanoinks not only for circuit-forming materials^23–26^ but also die attach materials^27–29^. However, since silver is an expensive precious metal and the price of silver ingots substantially fluctuates, the application range may be limited. Therefore, studies utilizing inexpensive copper as nanoinks for circuit-forming materials and bonding materials instead of silver nanoparticles have expanded^30–34^ because the thermal conductivity of bulk copper is 398 W·m^−1^·K^−1^. To date, various methods, such as hydrazine reduction^2,35,36^, polyol reduction^37–41^, thermal decomposition^42^, and electrochemical reduction^30^, have been proposed for the synthesis of copper nanoparticles (NPs) with low-temperature sintering ability. Despite extensive efforts by researchers, the above-mentioned methods require the use of polymers and surfactants to control the diameter of copper NPs and prevent aggregation in organic solvents. Producing copper NPs with a low cost on an industrial scale for practical applications is potentially problematic because the utilization of surfactants and organic solvents results in waste disposal issues. Many sintering copper pastes for intermetallic bonding that use copper NPs obtained via the above synthetic method have recently been reported; however, unlike a conventional sintering atmosphere, such as air or nitrogen, which is used for solder, a conventional intermetallic bonding material, a slightly reducing atmosphere is required for sintering of copper NPs^30,37^. The lower oxidation resistance, lower storage stability, and higher sintering temperature of copper NPs than those of the corresponding silver NPs are also fundamental problems for practical applications of copper-NP-based paste technology.

According to Pawlow’s equation^43^, the apparent sintering temperature of metal NPs depends on the particle size. Burke^44^ and Kusama^45^ theoretically and experimentally demonstrated, respectively, that polycrystalline particles show lower sintering behavior than the corresponding metal particles. Based on this information, we consider that copper NPs with a mean particle diameter of several tens of nanometers with a small crystallite size (*C*_*s*_) in the NP core could be expected to have an excellent low-temperature sintering ability, high oxidation resistance, and high storage stability. To synthesize specifically designed copper NPs, a polymer and surfactant that inhibit sintering should not be used as the anti-aggregation and anti-oxidation agents. The solvent is limited to water due to environmental concerns, and the NPs can be synthesized at room temperature. Here, we report that copper NPs with excellent low-temperature pressureless sintering properties under a nitrogen atmosphere were successfully obtained by an ambient aqueous-phase reductive synthesis using a water-soluble copper complex as a raw material. The copper NP-based pastes obtained exhibit a low resistivity and ultrahigh bonding ability as a die attach material applicable for practical usage.

## Results and Discussion

### Ambient aqueous-phase synthesis of copper NPs

**Figure 1:**
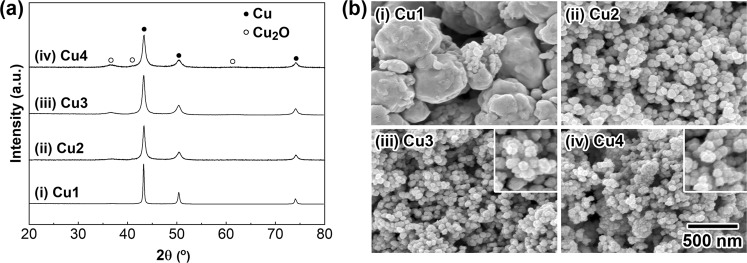
Crystal structure analysis and morphological observation of copper NPs. (**a**) XRD patterns of the copper NPs obtained with different molar ratios of [NTA H 2Na]/[Cu(OH)_2_]: (i) **Cu1**: 0; (ii) **Cu2**: 1.69; (iii) **Cu3**: 2.0; and (iv) **Cu4**: 2.4. All reactions were carried out at 25 °C for 1 h. (**b**) SEM images of the copper NPs formed by the changing the molar ratio of [NTA H 2Na]/[Cu(OH)_2_]: (i) **Cu1**: 0; (ii) **Cu2**: 1.69; (iii) **Cu3**: 2.0; and (iv) **Cu4**: 2.4. The scale bar shown in (iv) is the same for (i)–(iii). Insets in (iii) and (iv) are the 2-fold magnified images.

To obtain copper NPs with a mean particle diameter of several tens of nanometers, we focused on a reduction of an *in situ*-prepared organocopper reagent in an aqueous-phase system. After optimizing the reaction conditions, a reduction method enabled us to prepare a large quantity of specifically designed copper NPs with precisely controlled particle size and shape in an atmospheric aqueous solution system. The established reaction procedure is as follows. Initially, a water-soluble copper precursor (copper(II) ions: 0.27 mol/L) was prepared by mixing nitrilotriacetic acid disodium salt (NTA H 2Na) and Cu(OH)_2_ powder in water at room temperature with stirring. Then, an aqueous solution of hydrazine monohydrate (4.9 mol/L) was rapidly added in one portion under atmospheric conditions to the precursor solution to obtain copper NPs. The resulting copper NPs were purified by dispersing them in water after centrifugation. To investigate the effect of the [NTA H 2Na]/[Cu(OH)_2_] molar ratio on the copper NP preparation, the ratio was changed in the range from 0 to 2.4. Figure 1(a,b) show the X-ray diffraction (XRD) patterns and scanning electron microscopy (SEM) images, respectively, of the solid particles that were formed by the hydrazine treatment with different molar ratios of [NTA H 2Na]/[Cu(OH)_2_] ((i) 0, (ii) 1.69, (iii) 2.0, and (iv) 2.4). All diffraction peaks in Fig. 1(a,i) can be assigned to the formation of an *fcc*-type metallic copper crystal structure (JCPDS No. 00-4-836), indicating the obtained copper particles were in a single phase. In contrast, when NTA H 2Na was used as the chelating agent, metallic copper was the predominant phase, and a slight amount of Cu_2_O phase (JCPDS No. 00-5-667) was observed (Fig. 1(a)(ii)–(iv)). The diffraction peaks due to the Cu_2_O phase became stronger as the [NTA H 2Na]/[Cu(OH)_2_] molar ratio increased from 1.69 to 2.4. Partial surface oxidation of copper NPs during the purification process might cause the formation of the Cu_2_O phase. The SEM images of the as-prepared particles are shown in Fig. 1(b). The copper particles obtained in the absence of NTA were submicrometer in size with an irregular, coagulated shape (Fig. 1(b)(i); these particles are abbreviated as **Cu1**). Based on the XRD patterns (Fig. 1(a)(ii),(iii), and (iv)), the particles with a uniform spherical shape, as seen in Fig. 1(b)(ii),(iii), and (iv) and abbreviated as **Cu2**, **Cu3**, and **Cu4**, respectively, could be assigned as metallic copper NPs. The results suggest that NTA plays an important role in controlling the size and shape of copper NPs, and **Cu1**, which was obtained without using NTA, was not applicable for copper nanopastes due to its large diameter. Characterization of the frontier surfaces of **Cu3** by diffuse reflectance infrared Fourier transform (DRIFT) and X-ray photoelectron spectroscopy (XPS) methods revealed that NTA adsorbed on the surfaces, and thermogravimetric and differential thermal analysis (TG-DTA) indicated 2.1 wt% NTA was present (*see*, supplementary information (SI)). Yonezawa *et al*. reported that a small amount of low-molecular-weight gelatin on a Cu_2_O layer on the surface of copper NPs prevented further oxidation of the copper NPs^36^. Based on our results, the NTA on the surface of the copper NPs also prevents oxidation of the copper NPs in the present study.

**Figure 2:**
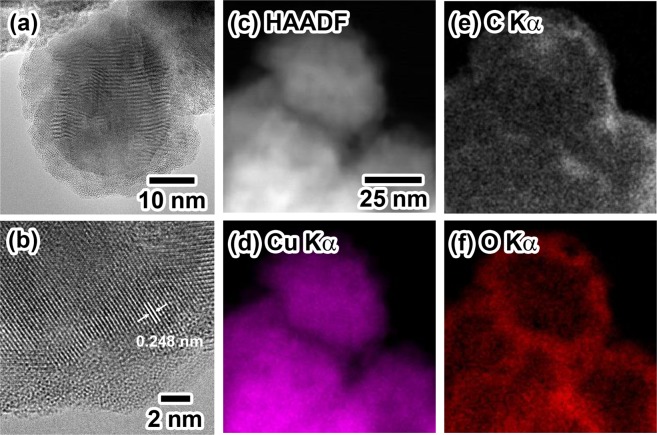
Characterization of the surface and internal structures of **Cu3**. HR-TEM and HAADF-STEM images of **Cu3**. (**a**) An HR-TEM image of **Cu3**; (**b**) A close-up image of **Cu3** shown in (**a**,**c**) A HAADF scanning image of the EDS analysis area. (**d**) copper *K*_α1_; (**e**) carbon *K*_α1_; (**f**) oxygen *K*_α1_.

Next, the average particle diameter (*D*_ave_) and standard deviation (σ) of **Cu2**, **Cu3**, and **Cu4** were determined by counting more than 150 NPs in the corresponding SEM images, and the diameters were 75.1 (13.0) nm, 59.2 (12.6) nm, and 51.4 (8.3) nm, respectively. The diameters decreased with an increase in the [NTA H 2Na]/[Cu(OH)_2_] molar ratio. The decrease in diameter resulted in an increase in the surface area of the copper NPs. The increase in the surface area might accelerate partial oxidation on the surfaces under atmospheric conditions. Furthermore, the surface roughness of the copper NPs (**Cu2-Cu4**) might also enhance partial oxidation, resulting in the formation of the Cu_2_O phase on the surface of the copper NPs. The increase in the peak intensities of the Cu_2_O phase with the decrease in *D*_ave_ observed in Fig. 1(a)(ii)–(iv) was in good agreement with the oxidation behavior on the surfaces. Here, the initial crystallite sizes (*S*_*i*_) of **Cu2**, **Cu3**, and **Cu4** before sintering were calculated using the half width of the (111) diffraction peak and Scherrer’s equation^46^ and were (ii) 15.5 nm, (iii) 14.0 nm, and (iv) 11.8 nm, respectively. The large difference between *D*_ave_ and *S*_*i*_ strongly suggests that the present copper NPs have an internal polycrystalline structure. Figure 2 presents the characterization results for the internal structure and surface state of **Cu3** obtained using a high-resolution transmission electron microscope (HR-TEM) and a high-angle annular dark field scanning TEM (HHADF-STEM) equipped with an energy dispersive X-ray spectroscopy (EDS) system. Figure 2(a) shows the HR-TEM images of **Cu3**, and lattice fringes and grain boundaries due to the formation of polycrystalline structures are clearly observed inside the particles. The image also suggests the existence of a rough shell structure on the surface of **Cu3** with a thickness of *ca*. 5 nm. Based on Fig. 1(a)(iii), the **Cu3** copper NPs contain some Cu_2_O phase. The detailed observation of the thin layers on the surfaces of **Cu3** revealed some lattice fringes in the layer, and the lattice interval of 0.248 nm could be assigned as the (111) plane of the Cu_2_O phase (Fig. 2(b)). Rietveld analysis of the XRD (Fig. 1(a)(iii)) pattern of **Cu3** revealed that that the weight ratio of the Cu_2_O/Cu was assigned as 17/83. The above results indicate that **Cu3** has a copper metal core and Cu_2_O shell structure, and NTA molecules were adsorbed on the Cu_2_O shell layer to prevent oxidation of the copper metal core. The STEM-EDS images shown in Fig. 2(e,f) are the mapping images of carbon and oxygen atoms, respectively. Both elements are distributed on the surfaces of the NPs and can be recognized as carbon atoms in the NTA molecules and oxygen atoms in the Cu_2_O phase and the NTA molecules. The images also support the formation of a unique NTA- and Cu_2_O-covered polycrystalline structure. This unique structure is the possible reason for the high oxidation resistance, long-term stability, and low-temperature sintering, and ultrahigh bonding abilities of the copper nanopastes mentioned in the following section. Details on the extensive efforts to clarity the effects of the chelation agent, reductant, and initial pH on the copper NP synthesis applicable to the present copper NP-based pastes will be reported elsewhere by the authors.

### Sintering behavior of the copper NPs

**Figure 3:**
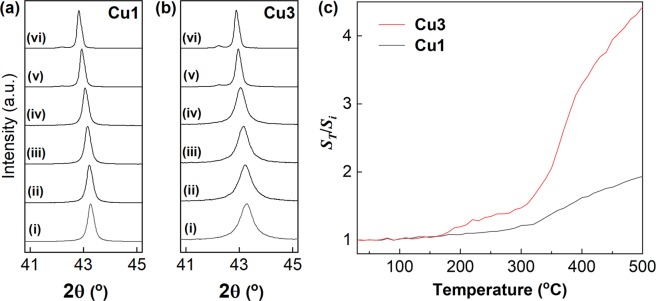
Sintering behavior of the copper NPs. (**a**) Change in the copper (111) XRD peak of **Cu1** (i) before heating; after heating at (ii) 100 °C, (iii) 200 °C, (iv) 300 °C, (v) 400 °C, and (iv) 500 °C. (**b**) The behavior of **Cu3**. The shift to lower angles with heating, which was observed for (**a**,**b**), is due to the thermal expansion of the crystal lattice of copper. (**c**) Relationship between *S*_*T*_/*S*_*i*_ and the sintering temperature (_*T*_) of **Cu1** and **Cu3**. *S*_*T*_/*S*_*i*_: crystallite size ratio at _*T*_ (*S*_*T*_: *C*_*s*_ at _*T*_).

To investigate the pressureless sintering ability of the present copper NPs, temperature-variable XRD measurements were carried out with pressureless heating. The experimental details are summarized in the Methods section. The copper (111) diffraction peaks of **Cu1** and **Cu3** at 30, 100, 200, 300, 400, and 500 °C are summarized in Fig. 3(a,b), respectively. Here, the peak shift to lower angles was due to thermal expansion of the crystal lattice with the increase in the temperature. For **Cu1**, the half width of the (111) diffraction peaks slightly sharpened with the increase in the heating temperature. The lattice intervals of the (111) planes at 30 °C and 500 °C were calculated as 0.209 nm and 0.211 nm, respectively. The expansion was good agreement with the reported thermal expansion of Cu^47^. In contrast, the corresponding half width of **Cu3** drastically narrowed under the same treatment, and the *C*_*s*_ of **Cu3**, which has a much smaller *D*_*ave*_ than **Cu1**, increased from 14.0 nm to 62.1 nm as the temperature increased from 30 °C to 500 °C. To clarify this behavior, Fig. 3(c) summarizes the relationship between the *C*_*s*_ ratio, *S*_*T*_/*S*_*i*_, and the sintering temperature (_*T*_). Here, *S*_*T*_ represents *C*_*s*_ at _*T*_ °C. The temperature range was 30 °C to 500 °C, and the heating rate was 1 °C/min. The interval to measure the XRD profile was 10 °C. All the XRD patterns were taken after holding the samples at _*T*_ °C for 1 min. In the case of **Cu3** (*S*_***i***_ of **Cu3**: 14.0 nm), a rapid increase in the *S*_*T*_/*S*_*i*_ value was observed at *ca*. 170 °C, and the *S*_*T*_/*S*_*i*_ value gradually increased from 200 to 300 °C. It is worth noting that such a rapid increase in the value at *ca*. 170 °C was not observed in the case of **Cu1** (*S*_*i*_ of **Cu1**: 37.0 nm). In addition, a further increase in the sintering temperature above 300 °C resulted in an increase in the *S*_*T*_/*S*_*i*_ values for both **Cu3** and **Cu1**. In previous studies^28^, copper pastes were heat-treated at temperatures higher than 300 °C to fabricate the corresponding sintered copper electrodes by adhering copper particles through decomposition of organic modifiers on surfaces and grain boundary diffusion. Based on the results shown in Fig. 3(c), the increase in the *S*_*T*_/*S*_*i*_ values in the range from 170 °C to 300 °C, which was only observed for **Cu3**, could indicate growth of *C*_*s*_ by diffusion on the rough surfaces of **Cu3** to connect the **Cu3** NPs. This behavior, that is, the increase in the *S*_*T*_/*S*_*i*_ value of **Cu3** at low temperature, is the most plausible reason for the remarkable low-temperature pressureless sintering ability of the **Cu3**-based nanopastes, as mentioned below.

### Sintering behavior of the copper nanopaste and resistivity of the resulting copper electrodes

**Figure 4:**
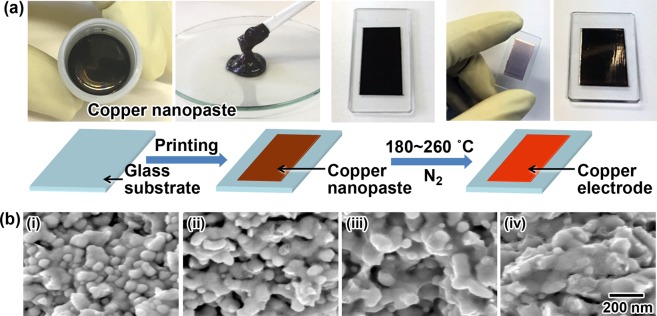
Preparation of copper electrodes using the nanopaste and characterization of the resulting electrodes. (**a**) A schematic illustration of the preparation of copper electrodes using the present copper nanopaste; (**b**) SEM images of the copper electrodes made from the nanopaste on a glass substrate and pressureless sintered at (i) 180 °C, (ii) 200 °C, (iii) 230 °C, and (iv) 260 °C under an N_2_ atmosphere for 30 min.

For the nanopaste copper NPs, **Cu3** was used due to its low-temperature sintering property. Details about the preparation of the copper nanopaste are shown in the Methods section. To investigate the sintering characteristics, the copper nanopaste was printed on a glass substrate with a thickness of 55 µm and pressureless sintered at 180, 200, 230, and 260 °C for 30 min under a nitrogen atmosphere to obtain copper electrodes. Figure 4 summarizes the printing process of the copper electrodes and the characterization results for the copper electrodes on a glass substrate. A schematic illustration and photos of each step in the preparation of the copper electrodes using the copper nanopaste are shown in Fig. 4(a). As shown in Fig. 4(a) on the right, a metallic-colored copper electrode is readily fabricated on a glass substrate by simple printing and pressureless sintering at 200 °C for 30 min. The resistivity of the copper electrodes pressureless sintered at 180 °C, 200 °C, 230 °C, and 260 °C for 30 min was 68 µΩ·cm, 16 µΩ·cm, 15 µΩ·cm, and 10 µΩ·cm, respectively. The resistivity decreased with the increase in the sintering temperature, and a remarkably low resistivity was achieved with low-temperature sintering in comparison to that reported in previous studies^30^. Furthermore, thick and strong copper electrodes (26 µm at 180 °C; 20 µm at 260 °C), which are applicable for practical usage, were readily constructed on glass substrates. To compare the sintering behavior of the **Cu3** powder and the **Cu3**-based nanopaste, the *C*_*s*_ values of the nanopaste at _*T*_ (*S*_*pT*_) were calculated from the half width of the (111) XRD peak at _*T*_ using Scherrer’s equation. The *S*_*pT*_ values at _*T*_ = 180 °C, 200 °C, 230 °C, and 260 °C were 26.3 nm, 32.8 nm, 40.8 nm, and 42.1 nm, respectively, and the corresponding *S*_*pT*_/*S*_*i*_ of **Cu3** values were calculated to be 1.88, 2.34, 2.91, and 3.01, respectively. In contrast, the *S*_*T*_/*S*_*i*_ of **Cu3** values, which indicate the sintering behavior of the **Cu3** powder at _*T*_ = 180 °C, 200 °C, 230 °C, and 260 °C, were 1.11, 1.19, 1.28, and 1.38, respectively (*see* Fig. 3(c)). Note that there is a large difference between the *S*_*pT*_/*S*_*i*_ of **Cu3** and *S*_*T*_/*S*_*i*_ of **Cu3** values at their respective _*T*_ values. The results clearly indicate that the low-temperature pressureless sintering ability to grow crystallite **Cu3** was drastically enhanced by the pasting. Figure 4(b)(i)–(iv) are the SEM images of the corresponding copper nanopaste after pressureless sintering at (i): 180 °C, (ii): 200 °C, (iii): 230 °C and (iv) 260 °C for 30 min. The SEM images show that an increase in the sintering temperature enhanced the melting of the surfaces of the NPs, which was followed by connections forming between the NPs. The images also shown that the domain sizes increase with the increase in the sintering temperature. However, the domain sizes in the SEM images were much larger than the corresponding *S*_*pT*_ because all the *S*_*pT*_ values in the range from 180 to 260 °C were several tens of nanometers. This result indicates that each domain in the copper electrodes still has a polycrystalline structure. The results also support the conclusion that the sintering observed in the present study mainly proceeds by the diffusion of copper atoms on the rough surfaces of **Cu3** to connect the **Cu3** NPs. Controlling the surface morphology of metal pastes might be a powerful technique to introduce a low-temperature sintering ability. Next, the sintering behavior of the **Cu3**-based nanopastes was compared with the corresponding **Cu3** powder. As mentioned above, **Cu3** has a Cu_2_O shell on its surface. On the other hand, the copper nanopastes contain triethanolamine (TEA) as a dispersion medium. Here, TEA reduced the heating requirement^48^. In the case of the present copper nanopaste, reduction of the Cu_2_O shell on the surface of the copper NPs by the TEA in the nanopaste might strongly enhance the sinterability of these copper NPs at low temperatures.

### Preparation of electric circuits on flexible films using the copper nanopaste

**Figure 5:**
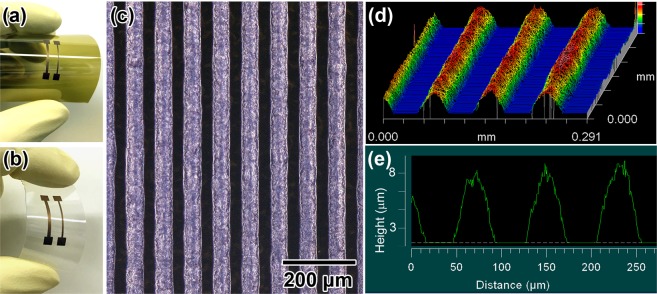
Fabrication of copper electric circuits on flexible films by screen printing the copper nanopastes. (**a**) Copper electrodes on a PI film with bending. (**b**) Copper electrodes on a PEN film. (**c**) An optical microscopic image of the copper electric circuits prepared by screen printing on a PI film using a fine pattern screen mask (L/S: 40/40 (μm/μm)). (**d**) A 3D image of the copper electric circuits shown in (**c**). (**e**) A depth profile of the copper electric circuits. The height of the circuits was approximately 8 μm. The printed copper nanopastes on the films were pressureless sintered at 200 °C under a N_2_ atmosphere for 30 min.

Copper nanopaste-based electric electrodes on polyimide (PI) and polyethylene terephthalate (PEN) flexible films were printed by the procedure shown in the Methods section. Figure 5(a,b) show photos of the copper electrodes on PI and PEN flexible films, respectively, which were prepared by screen printing and pressureless sintering at 200 °C under a N_2_ atmosphere for 30 min. Cracks and detachment of the electrodes from the films were not observed after repeated bending of the films (also *see*, Fig. S4 in SI), and the copper nanopaste was found to be a widely applicable wiring material for not only on a glass substrate but also plastics films. Next, copper electric circuits were screen printed on a PI film using a fine pattern screen mask. The line (L) and space (S) of the mask, L/S, were 40/40 μm/μm. The printed films were pressureless sintered at 200 °C under a N_2_ atmosphere for 30 min to obtain conductive circuits. The resulting L/S values and thickness of the copper electric circuits after sintering were measured by an optical microscope. Figure 5(c) exhibits an optical microscopic image of the copper electric circuits. Uniform and straight copper electrodes were fabricated on the PEN film. The average L/S could be assigned as 50/30 μm/μm. The 3D and depth profiles are also shown in Fig. 5(d,e), respectively. The average height was 8 μm, and thick copper electrodes were readily obtained by a simple printing process.

### Share strength of die attachment using the copper nanopaste

**Figure 6:**
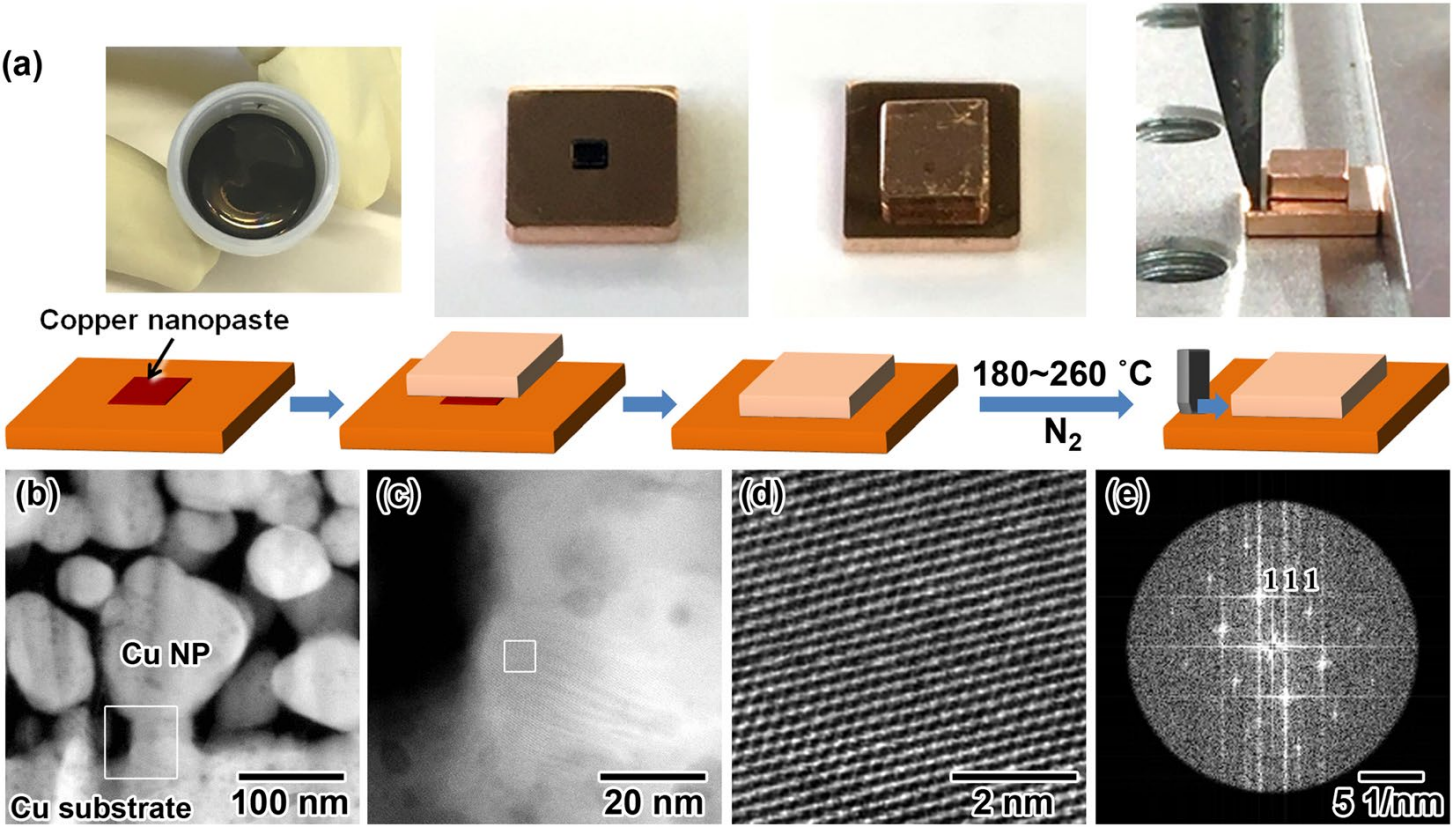
Adhesion of the copper substrates with use of the copper nanopaste and the share strength of the die attach material. (**a**) Schematic and photo images for the preparation of a model IC chip-mounted die with use of the copper nanopaste and the die share stress measurement. (**b**) An HAADF-STEM image of the adhesion between copper NPs on the copper substrate. (**c**) A magnified image of the binding position between a copper NP and the copper substrate. The white-colored solid square shown in (**b**) is the magnified position. (**d**) An HAADF-STEM image to observe the crystallinity of the sintered position. The white-colored solid square exhibited in (**c**) is the magnified position. (**e**) A Fourier transfer image of (**d**). The bright spots could be assigned as (111) diffraction of Cu.

To investigate the performance of the **Cu3**-based copper nanopaste as a die attach material, the copper nanopaste was printed on an oxygen-free copper substrate (5 × 5 × 1 (mm)) in a 1 mm square using a metal screen mask, and then, an oxygen-free copper plate (3 × 3 × 1 (mm)) was mounted on the paste as a model IC chip. The resulting IC-mounted model substrate with a die attach structure was pressureless sintered at 180 °C, 200 °C, and 260 °C for 30 min under a N_2_ atmosphere. Figure 6(a) illustrates a schematic and images of the preparation of the mounted die using the copper nanopaste and the die share stress measurement procedure. The resulting share strength, which was measured by an XYZTEC bond tester, was 25 MPa, 39 MPa, and 38 MPa, respectively. The share strengths at 200 °C and 260 °C were approximately the same, indicating 200 °C was a sufficient temperature to ensure a stable adhesion between copper substrates. It is worth noting that the share strength observed here is much higher than 35 MPa, and copper nanopastes with high performance by pressureless sintering at 200 °C have not been reported thus far^15–17,49^. This result means that the present copper nanopaste has an ultrahigh bonding ability and is practically applicable as a die attach material with a low-temperature sintering ability. Figure 6(b–e) shows the results of the HAADF-STEM observations of the bonding state between the copper NPs and the copper substrate. The inter-fringe distance of 0.206 nm in Fig. 6(d) was assigned as the distance of the (111) planes of the metallic copper phase. The results confirmed that metallic bonding with uniform lattice fringes spontaneously occurred between the copper particles and the copper substrate by sintering despite the absence of a reducing gas, such as hydrogen or formic acid (Fig. 6(d)). Although the remarkable low-temperature pressureless sintering mechanism to form single-crystalline metallic bonding at 200 °C under a nitrogen atmosphere was unclear, a highly crystalline, connected structure with uniform lattice fringes between the copper NPs and the copper substrate was the reason for the unprecedented ultrahigh bonding property with a strength of 38 MPa achieved in the present study.

## Methods

### Reagents

Unless otherwise noted, all reagents were used as received. Water was doubly distilled, deionized, and filtered prior to use. Reagent grade nitrilotriacetic acid disodium salt (NTA·H·2Na, 99.5%) was purchased from CHELEST Corporation. Copper hydroxide (Cu(OH)_2_, 90.0%, Cat. No: 031-04215) was purchased from Wako Pure Chemical Industries, Co., Ltd. Sodium hydroxide (97.0%, Cat. No: 198-13765), hydrazine monohydrate (98.0%, Cat. No: 18383-00), ethanol (99.5%, Cat. No: 14033-00), triethanolamine (TEA, 99.0%, Cat. No: 40268-00), and dehydrated methanol (99.8%, Cat. No: 25506-25) were purchased from Kanto Chemical Co., Inc. 3-Glycidoxypropyltrimethoxysilane was purchased from Shin-Etsu Chemical Co., Ltd.

### Ambient aqueous-phase synthesis of copper NPs

The synthetic procedure for the preparation of copper NPs via ambient aqueous-phase synthesis is as follows. Initially, in a round-bottomed 1 L flask, aqueous NaOH (2.8 mol/L, 43 mL) and NTA·H·2Na, (0, 0.60, 0.71, and 0.84 mol/L, 340 mL) solutions were mixed together with stirring at room temperature under an air atmosphere. Then, Cu(OH)_2_ powder (11.8 g, 0.12 mol) was added to the mixed aqueous solution and dissolved in the solution with stirring under the same environment. The resulting molar ratios of [NTA·H·2Na]/[Cu(OH)_2_] were 0, 1.69, 2.0, and 2.4. Here, in the absence of NTA, Cu(OH)_2_ powder was remained in the precursor solution. Next, an aqueous solution of hydrazine monohydrate (4.9 mol/L, 98 mL) at room temperature was added in one portion with stirring (300 rpm). The resulting mixture was further stirred for 1 h at the same temperature. The obtained dark purple solid particles and the aqueous phase were separated by centrifugation (10,000 G, 30 min), and the sediments were washed two times with water and three times with ethanol by dispersal and centrifugation to collect the copper NPs as an ethanol slurry (70 wt.% Cu). For the characterization of the copper NPs, the slurry was dried under reduced pressure to obtain the copper NPs as a powder.

### Preparation of the copper NP-based pastes

The copper NP-based pastes used in the present study were prepared as follows. TEA (5.29 mL) was added to the obtained ethanol slurry of copper NPs (30.0 g) at room temperature. The ethanol in the mixture was completely removed under reduced pressure with stirring. Then, to prevent to form cracks, a methanolic solution of 3-glycidoxypropyltrimethoxysilane (63 wt.%, 2.18 mL) was added to the mixture to obtain the copper NP-based pastes (80 wt.% Cu). The obtained copper paste was stable at room temperature for 1 day and was confirmed to be stable for 1 year by cryopreservation. The viscosity of the copper paste was 45 Pa·s (10/s), 7 Pa·s (100/s).

### Fabrication of copper NP-based pattern electrodes on glass substrates for the resistivity evaluation

Copper-pattern electrodes on a glass substrate (OA-10G: 30 mm × 15 mm; *t* = 0.7 mm, Nippon Electric Glass Co., Ltd.) were prepared by the following procedure. Initially, the copper NP-based pastes were coated on the glass substrates by screen printing using a metal screen mask manufactured by Tokyo Process Service Co., Ltd.. The thickness of the pastes was adjusted to 55 μm. Then, the casted substrates were pressureless sintered at 180, 200, 230, and 260 °C under an N_2_ atmosphere for 30 min. The resistivity of the resulting sintered substrates with different sintered temperatures was measured by a four-point probe method.

### Preparation of copper NP-based pattern electrodes on flexible films

Copper-electric circuits on flexible films were obtained by the following procedure. As flexible films, a polyimide film (UPIREX, Ube Industries Ltd., *t* = 25 μm) and polyethylene terephthalate film (Teonex Q65FA, TEIJIN Ltd., *t* = 200 μm) were chosen. The copper NP-based pastes were screen printed on flexible films using a fine pattern screen mask (high density mesh ST 500, emulsion thickness: 15 μm) manufactured by Tokyo Process Service Co., Ltd.. The line (L) and space (S) of the mask (L/S) were 40/40 μm/μm. The printed films were pressureless sintered at 200 °C under a N_2_ atmosphere for 30 min to obtain the conductive circuits. The resulting L/S values and thickness of the copper electrodes after sintering were measured by an optical microscope.

### Preparation of a copper-bonded body for the share strength measurement

The oxygen-free copper plate (i-ject Co., Ltd., 5 × 5 mm; *t = *1 mm) was polished with a rotary polishing machine (mesh size: # 4000) and a copper plate with a fresh and smooth surface was used for the test. The copper NP-based paste was printed in a 1 mm square on a fresh oxygen-free copper plate using a metal screen mask (Tokyo Process Service Co., Ltd.). Then, the paste was sandwiched by an oxygen-free copper plate (i-ject Co., Ltd., 3 × 3 mm; *t* = 1 mm). The resulting bonded bodies were sintered at 180, 200, 230, and 260 °C under a pressureless and N_2_ atmosphere for 30 min for adhesion.

### Characterization equipment

The pH values during the copper NP synthesis were monitored using a pH electrode (HORIBA Ltd., 9615S-10D). The size and shape of the copper NPs were observed by an FEI XL 30SFEG scanning electron microscope (SEM) with an acceleration voltage of 5 kV. High-resolution transmission electron microscopic (HR-TEM) and high-angle annular dark field scanning TEM (HAADF-STEM) images were taken by an FEI TITAN 80–300 instrument at 200 kV and a JEOL ARM200F instrument at 200 kV equipped with an EDS analyzer, respectively. The diffuse reflectance infrared Fourier transform (DRIFT) for the characterization of the surface properties of the copper NPs was measured with a Thermo Scientific, Inc. NICOLET 6700 spectrometer equipped with an ATR system under a nitrogen atmosphere. The surface state of the copper NPs was evaluated by ULVAC-PHI PHI 5000 VersaProbe II X-ray photoelectron spectroscopy (XPS) with AlKα radiation (50 W, 15 kV). The crystal system and crystallite size of the copper NPs were measured by a Rigaku Intelligent X-ray diffraction (XRD) SmartLab system equipped with a PILATUS3 R 100 K detector using CuKα radiation (40 kV, 40 mA). Temperature-variable XRD measurements were also carried out by the SmartLab system under a nitrogen atmosphere for the evaluation of the sintering temperature dependency of the copper NPs. The temperature range and heating speed were 30–500 °C and 1 °C/min, respectively. The XRD patterns were taken at intervals of 10 °C in the range from 30 to 500 °C. The heating rate was fixed at 1 °C/min, and the holding time before the measurement was 1 min. The crystallite size of the NPs was calculated by Scherrer’s equation^46^ (Scherrer constant: 1.33) using the half width of the (111) diffraction peak. Thermogravimetric and differential thermal analysis (TG-DTA) of the copper NPs was measured using a Rigaku Thermo plus EVO2 TG8121 high-temperature model at a heating rate of 1 °C/min in a nitrogen atmosphere. For the electrical conductivity of the copper-pattern electrodes, the resistivity value was measured using a Mitsubishi Chemical Analytech Loresta-GX MCP-T 700 instrument with a four-point probe method. The share strength of the joined body was measured and calculated by an XYZTEC bond tester Condor Sigma at a shear tool speed of 50 μm s^−1^. The bonding states between the copper paste and the copper plate of the bonded body were observed using a HAADF-STEM system.

## Conclusions

In the present study, we designed and synthesized copper NPs that are applicable for practical copper nanopastes for the first time, and the resulting nanopastes exhibited remarkably low-temperature pressureless sintering behavior for the fabrication of thick copper electrodes on not only glass substrates but also flexible films, such as PI and PEN. Furthermore, the copper nanopastes thus obtained were successfully applied as a die attachment material. The strength of the bonding between a model IC chip and a copper substrate was 39 MPa after pressureless sintering at 200 °C for 30 min. The remarkable ultrahigh bonding strength and low-temperature pressureless sintering ability of this material make the material applicable as a promising alternative to soda alloys for constructing SiC- and GaN-based IC chips for next-generation power devices. Our copper NP-based nanopastes have the following advantages: (i) the copper NPs can be readily obtained in a large quantity from an aqueous solution system under ambient atmospheric conditions without the use of expensive production equipment, such as an autoclave and heater; (ii) the water-based copper NP preparation system does not require the use of organic solvents, surfactants, and dispersants, resulting in a system free from waste, environmental, and production-cost problems; and (iii) the copper NPs have a polycrystalline copper metal core and a thin NTA-modified Cu_2_O shell structure. The unique Cu_2_O-covered polycrystalline structure resulted in high oxidation resistance, long-term stability and low-temperature pressureless sintering and ultrahigh bonding abilities into the copper nanopastes. Our established water-based ambient NP synthesis through mild reduction of an *in situ*-prepared metal complex using a chelating agent might become a promising and powerful technique for the production of key materials essential for sustainable progress in PE technology as well as next-generation power device fabrication processes.

## Supplementary information

Supplementary Information

## References

[1] Khan S, Lorenzelli L, Dahiya RS (2015). Technologies for Printing Sensors and Electronics Over Large Flexible Substrates: A Review. IEEE Sens. J..

[2] Kamyshny A, Magdassi S (2014). Conductive Nanomaterials for Printed Electronics. Small.

[3] Wu W (2017). Inorganic Nanomaterials for Printed Electronics: A Review. Nanoscale.

[4] Perelaer J (2010). Printed Electronics: The Challenges Involved in Printing Devices, Interconnects, and Contacts Based on Inorganic Materials. J. Mater. Chem..

[5] Xue Q (2017). Facile Synthesis of Silver Nanowires with Different Aspect Ratios and Used as High-Performance Flexible Transparent Electrodes. Nanoscale Res Lett.

[6] Zhang SF (2018). Highly Conductive, Flexible and Stretchable Conductors Based on Fractal Silver Nanostructures. J. Mater. Chem. C.

[7] Khan Y (2016). Flexible Hybrid Electronics: Direct Interfacing of Soft and Hard Electronics for Wearable Health Monitoring. Adv. Funct. Mater..

[8] Chang JS, Facchetti AF, Reuss R (2017). A Circuits and Systems Perspective of Organic/Printed Electronics: Review, Challenges, and Contemporary and Emerging Design Approaches. IEEE Journal on Emerging and Selected Topics in Circuits and Systems.

[9] Li X, Andersson H, Sidén J, Schön T (2018). Soldering Surface Mount Components on Screen-printed Ag Patterns on Paper and Polyimide Substrates for Hybrid Printed Electronics. Flexible and Printed Electronics.

[10] Arrese, J. *et al*. Flexible Hybrid Circuit Fully Inkjet-printed: Surface Mount Devices Assembled by Silver Nanoparticles-based Inkjet ink. *J*. *Appl*. *Phys*. **121**, 104904-1-104904-9 (2017).

[11] Matsuhisa N (2017). Printable Elastic Conductors by *in situ* Formation of Silver Nanoparticles from Silver Flakes. Nature Materials.

[12] Sakamoto S, Nagao S, Suganuma K (2013). Thermal Fatigue of Ag Flake Sintering Die-attachment for Si/SiC Power Devices. J. Mater. Sci. Mater. Electron..

[13] Siow KS (2012). Mechanical Properties of Nano-silver Joints as Die Attach Materials. J. Alloys Compd..

[14] Bai JG, Calata JN, Lu G-Q (2007). Processing and Characterization of Nanosilver Pastes for Die-Attaching SiC Devices. IEEE Transactions on Electronics Packaging Manufacturing.

[15] Peng P (2015). Joining of Silver Nanomaterials at Low Temperatures: Processes, Properties, and Applications. ACS Appl. Mater. Interfaces.

[16] Khazaka R, Mendizabal L, Henry D (2014). Review on Joint Shear Strength of Nano-Silver Paste and Its Long-Term High Temperature Reliability. J. Electron. Mater..

[17] Kewei X, Calata JN, Hanguang Z, Ngo KDT, Guo-Quan L (2013). Simplification of the Nanosilver Sintering Process for Large-Area Semiconductor Chip Bonding: Reduction of Hot-Pressing Temperature Below 200 °C. IEEE Transactions on Components, Packaging and Manufacturing Technology.

[18] Sakamoto S, Sugahara T, Suganuma K (2012). Microstructural Stability of Ag Sinter Joining in Thermal Cycling. J. Mater. Sci. Mater. Electron..

[19] Bai JG, Lu GQ (2006). Thermomechanical Reliability of Low-Temperature Sintered Silver Die Attached SiC Power Device Assembly. IEEE Transactions on Device and Materials Reliability.

[20] Suganuma K (2012). Low-temperature Low-pressure Die Attach with Hybrid Silver Particle Paste. Microelectronics Reliability.

[21] Manikam VR, Kuan Yew C (2011). Die Attaceh Materials for High Temperature Applications: A Review. IEEE Transactions on Components, Packaging and Manufacturing Technology.

[22] Kisiel R, Szczepański Z (2009). Die-attachment Solutions for SiC Power Devices. Microelectronics Reliability.

[23] Yamada T (2016). Nanoparticle Chemisorption Printing Technique for Conductive Silver Patterning with Submicron Resolution. Nat. Comun..

[24] Aoshima K (2018). Unique Coexistence of Dispersion Stability and Nanoparticle Chemisorption in Alkylamine/Alkylacid Encapsulated Silver Nanocolloids. Sci. Rep..

[25] Balliu E (2018). Selective Laser Sintering of Inkjet-printed Silver Nanoparticle Inks on Paper Substrates to Achieve Highly Conductive Patterns. Sci. Rep..

[26] Shiokawa, D. *et al*. Development of a Silver Nanoparticle Ink for Fine Line Patterning using Gravure Offset Printing. *Jpn*. *J*. *Appl*. *Phys*. **56**, 05EA04-1-05EA04-4, (2017).

[27] Li M, Xiao Y, Zhang Z, Yu J (2015). Bimodal Sintered Silver Nanoparticle Paste with Ultrahigh Thermal Conductivity and Shear Strength for High Temperature Thermal Interface Material Applications. ACS Appl. Mater. Interfaces.

[28] Lin SK (2016). Nano-volcanic Eruption of Silver. Sci. Rep..

[29] Zhang H, Nagao S, Suganuma K, Albrecht H-J, Wilke K (2015). Thermostable Ag Die-attach Structure for High-temperature Power Devices. J. Mater. Sci. Mater. Electron..

[30] Abhinav KV, Rao RVK, Karthik PS, Singh SP (2015). Copper Conductive Inks: Synthesis and Utilization in Flexible Electronics. RSC Adv..

[31] Kim H-S, Dhage SR, Shim D-E, Hahn HT (2009). Intense Pulsed Light Sintering of Copper Nanoink for Printed Electronics. Appl. Phys. A.

[32] Magdassi S, Grouchko M, Kamyshny A (2010). Copper Nanoparticles for Printed Electronics: Routes Towards Achieving Oxidation Stability. Materials (Basel).

[33] Niittynen J, Sowade E, Kang H, Baumann RR, Mantysalo M (2015). Comparison of Laser and Intense Pulsed Light Sintering (IPL) for Inkjet-printed Copper Nanoparticle Layers. Sci. Rep..

[34] Chen X (2017). Hybrid Printing Metal-mesh Transparent Conductive Films with Lower Energy Photonically Sintered Copper/tin Ink. Sci. Rep..

[35] Dhas NA, Raj CP, Gedanken A (1998). Synthesis, Characterization, and Properties of Metallic Copper Nanoparticles. Chem. Mater..

[36] Yong Y (2017). Effect of Decomposition and Organic Residues on Resistivity of Copper Films Fabricated via Low-temperature Sintering of Complex Particle Mixed Dispersions. Sci. Rep..

[37] Jeong S (2008). Controlling the Thickness of the Surface Oxide Layer on Cu Nanoparticles for the Fabrication of Conductive Structures by Ink-Jet Printing. Adv. Funct. Mater..

[38] Lee Y, Choi JR, Lee KJ, Stott NE, Kim D (2008). Large-scale Synthesis of Copper Nanoparticles by Chemically Controlled Reduction for Applications of Inkjet-printed Electronics. Nanotechnology.

[39] Hokita Y, Kanzaki M, Sugiyama T, Arakawa R, Kawasaki H (2015). High-Concentration Synthesis of Sub-10-nm Copper Nanoparticles for Application to Conductive Nanoinks. ACS Appl. Mater. Interfaces.

[40] Ishizaki T, Watanabe R (2012). A New One-pot Method for the Synthesis of Cu Nanoparticles for Low Temperature Bonding. J. Mater. Chem..

[41] Park BK (2007). Synthesis and Size Control of Monodisperse Copper Nanoparticles by Polyol Method. J. Colloid Interface Sci..

[42] Togashi T (2017). *N*,*N*-Diethyl-diaminopropane-copper(ii) Oxalate Self-reducible Complex for the Solution-based Synthesis of Copper Nanocrystals. Dalton Trans.

[43] Pawlow P (1909). Über die Abhängigkeit des Schmelzpunktes von der Oberflächenenergie eines festen Körpers. Z. Phys. Chem..

[44] Burke JE, Turnbull D (1952). Recrystallization and GrainGrowth. Progress in Metal Physics.

[45] Kusama T (2017). Ultra-large Single Crystals by Abnormal GrainGrowth. Nat. Comun..

[46] Patterson AL (1939). The Scherrer Formula for X-Ray Particle Size Determination. Phys. Rev..

[47] Nix FC, MacNair D (1941). The Thermal Expansion of Pure Metals Copper, Gold, Aluminum, Nickel, and Iron. Physical Review.

[48] Kanie K (2011). Size-Controlled Hydrothermal Synthesis of Bismuth Sodium and Bismuth Potassium Titanates Fine Particles and Application to Lead-Free Piezoelectric Ceramics. Mater. Trans..

[49] Kahler J (2012). Sintering of Copper Particles for Die Attach. IEEE T. Comp. Pack. Man..

