# Supplementary material for: Ambient Aqueous-Phase Synthesis of Copper Nanoparticles and Nanopastes with Low-Temperature Sintering and Ultra-High Bonding Abilities
Source: Sci Rep. 2019 Jan 29;9:899. doi: 10.1038/s41598-018-38422-5 (PMC6349850; doi:10.1038/s41598-018-38422-5)
Supplement: Supplementary file 1 — Supplementary Information [file 41598_2018_38422_MOESM1_ESM.docx]

**Ambient Aqueous-Phase Synthesis of Copper Nanoparticles and Nanopastes with Low-Temperature Sintering and Ultra-High Bonding Abilities**

**Yoichi Kamikoriyama^1,2,^*, Hiroshi Imamura^2^, Atsushi Muramatsu^1^, and Kiyoshi Kanie^1,^***

^1^Tohoku University, Institute of Multidisciplinary Research for Advanced Materials, Sendai, 980-8577, Japan

^2^Mitsui Mining & Smelting Co., Ltd., Corporate Engineered Materials Sector R&D Center, Ageo, 362-0021 Japan

**Supplementary Information**

1. **Characterization of Cu3**
   1. **A Diffuse reflectance infrared Fourier transform (DRIFT) method

      Figure S1** exhibits a DRIFT profile of **Cu3**. An absorption can be assigned as a COOH moieties of NTA is slightly observed at *ca*. 1750 cm^-1^.


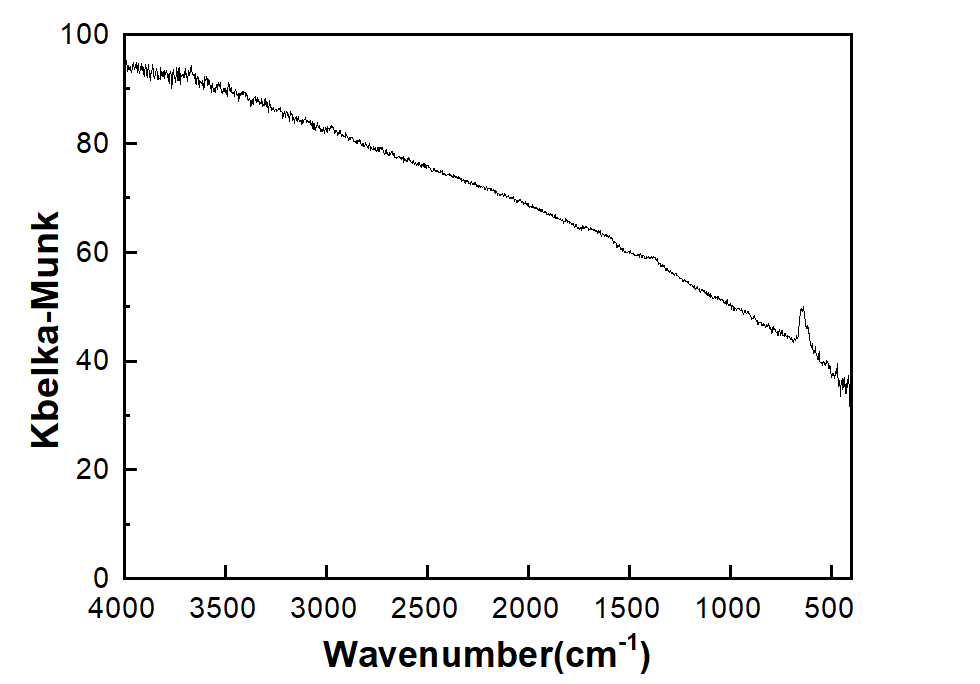


**Figure S1**. A DRIFT profile of **Cu3**.

- 1. **An X-ray photoelectron spectroscopic (XPS) observation**

**Figure S2** shows (a) C_1_*_s_* and (b) N_1_*_s_* XPS profiles of **Cu3** and NTA. Peaks due to NTS are clearly observed for **Cu3**.

**(a)**

**(b)**


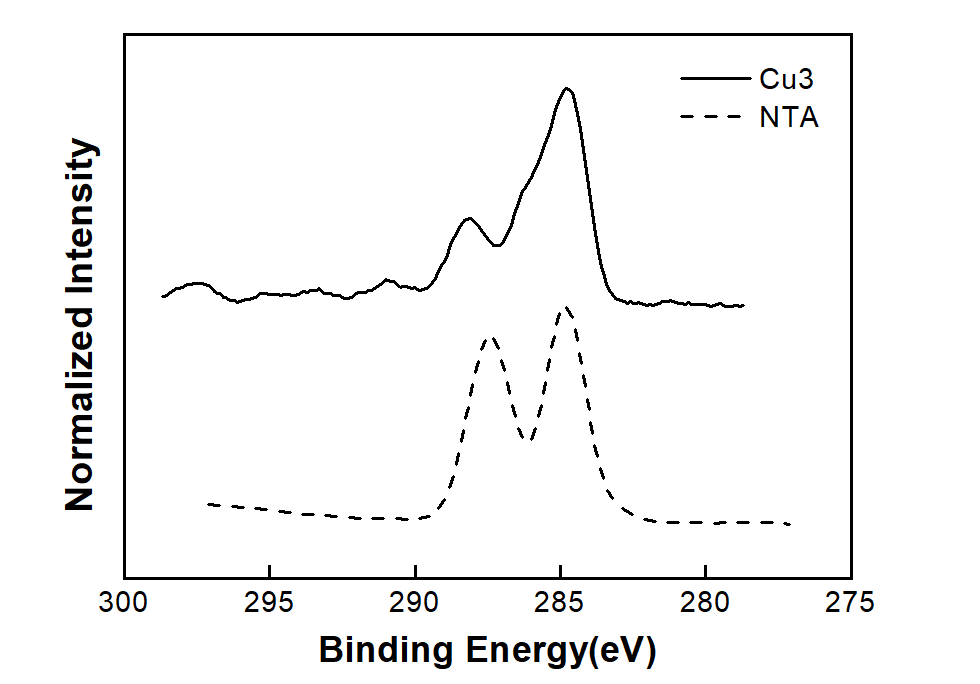

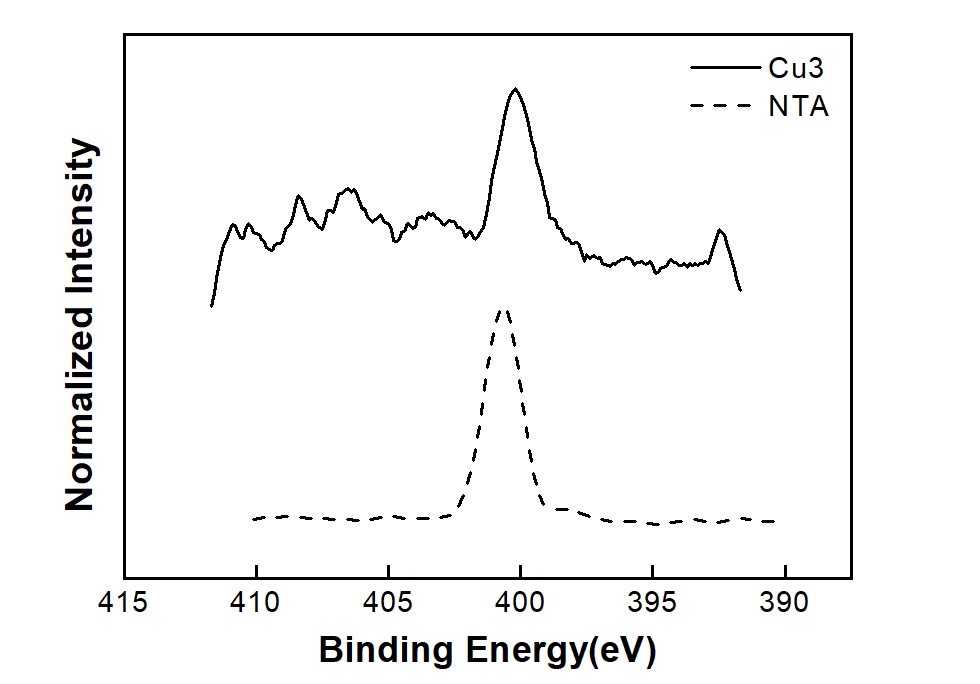


**Figure S2**. XPS profile of **Cu3** and NTA (a) C_1_*_s_*; (b) N_1_*_s_*.

- 1. **Thermogravimetric and Differential Thermal Analysis (TG-DTA) of Cu3

     Figure S3** exhibits TG-DTA curve of **Cu3.** Burning loss from ca. 180 °C to 250 °C due to degradation of NTA molecules on the surface of **Cu3** by heating.  **Figure S3**. TG-DTA curve of **Cu3** (heating rate: 5 °C/min).


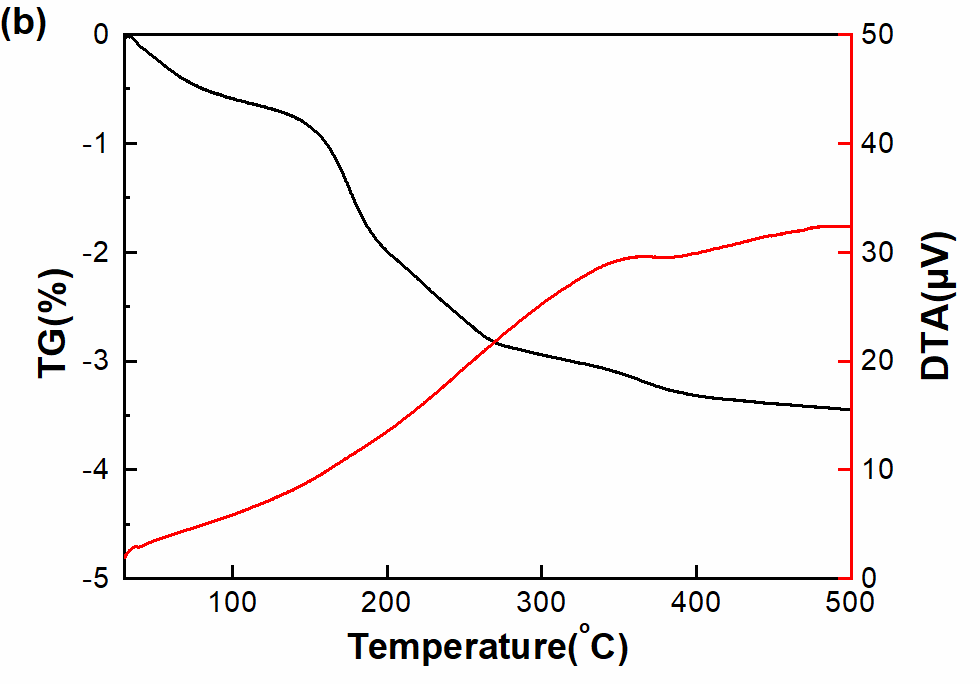


**2. Bending Test of Copper Nanopaste-based Electric Electrodes on a PI Flexible Film**

**Figure S4** exhibits an optical microscope image of electric electrodes on a PI flexible film prepared using **Cu3**-based nanopaste. The photo was taken after ten times bending. The bending diameter (φ) was fixed to 1 mm. None of cracks and detachment of the electrodes from the film was observed.


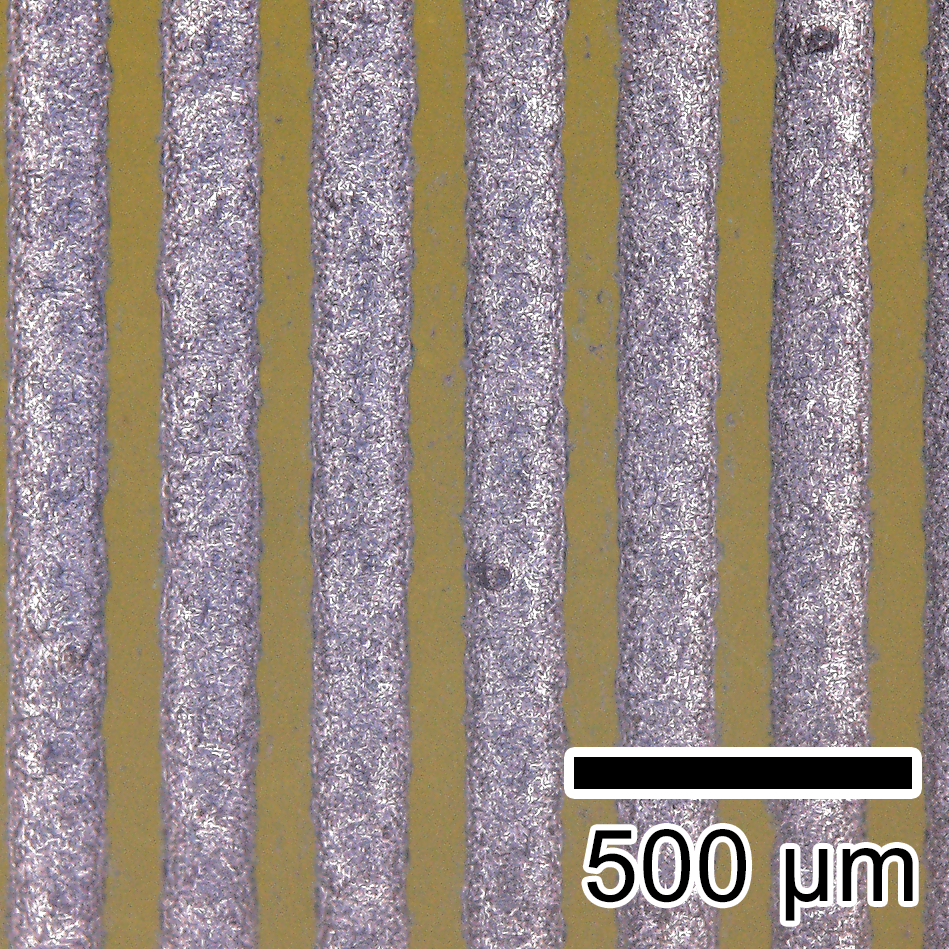


**Figure S4**. An optical microscope image of electric electrodes on PI flexible film prepared using **Cu3**-based nanopaste after ten times of inner and outer bending (φ = 1 mm).
